# Intensity-Modulated Radiotherapy for a Rendu-Osler-Weber Disease Patient with Recurrent Severe Epistaxis: A Case Report

**DOI:** 10.1155/2010/321835

**Published:** 2010-03-28

**Authors:** Maximilian Niyazi, Marco-Domenico Caversaccio, Patrick Dubach, Andreas Geretschläger, Andreas Arnold, Claus Belka, Daniel M. Aebersold, Norbert M. Blumstein

**Affiliations:** ^1^Department of Radiation Oncology, Ludwig-Maximilians-University München, Marchioninistr. 15, 81377 München, Germany; ^2^Department of Otolaryngology, Head and Neck Surgery, Inselspital, Bern University Hospital, University of Bern, CH-3010 Bern, Switzerland; ^3^Department of Radiation Oncology, Inselspital, Bern University Hospital, University of Bern, CH-3010 Bern, Switzerland

## Abstract

We present a case of a Rendu-Osler-Weber disease patient with recurrent life threatening epistaxis demanding multiple blood transfusions despite of repetitive endoscopic laser and electrocoagulations, endovascular embolisation, septodermoplasty, and long-term intranasal dressings. As alternative treatment modalities repeatedly failed and the patient became almost permanently dependent on nasal dressing, we performed a highly conformal intensity-modulated radiotherapy of the nasal cavity; a total dose of 50 Gy in 2 Gy single fractions was applied. The therapy was very well tolerated, no acute toxicities occurred. Two weeks after the last radiation dose had been applied, the nasal dressing could be removed without problems. Endoscopical control revealed an almost avascular white mucosa without any trace of bleeding spots; previously existing hemangiomas and crusts had disappeared. After a 1-year-follow up, the patient had no significant recurrent epistaxis.

## 1. Introduction

This study is a case report of a 69-year-old lady with Rendu-Osler-Weber disease who regularly attended the ENT outpatient ward of the Inselspital Bern because of recurrent severe epistaxis.

Hereditary hemorrhagic telangiectasia (HHT) or Rendu-Osler-Weber disease is an autosomal dominant disorder characterized by epistaxis, mucocutaneous telangiectases and visceral arteriovenous malformations which affects about 1 in 10,000 people [1]. There are hematologic, neurologic, pulmonary, dermatologic and gastrointestinal complications possible. Treatment is supportive and aims at preventing complications [2]. A treatment guideline for epistaxis in case of HHT recommends to do “as little as possible for as long as possible” [3] whereas treatment modalities include compression, topical antifibrinolitic therapy, laser coagulations, embolisations [4] and as an ultima ratio radiotherapy [5, 6].

## 2. Case Presentation

The 69-years-old lady presented in good performance and nutritional status. Because of the HHT-related recurrent severe epistaxis causing anemia she had undergone multiple sessions of blood transfusions, laser or elctrocoagulations, septal dermatoplastic interventions and transarterial embolisations in the past.

Additional medical diagnoses were hypertension with intermittent atrial fibrillation, left ventricular hypertrophy, pulmonary hypertension, visceral hypoperfusion, pyelonephritis, an old portal venous thrombosis, fatty liver, hyperuricemia and pulmonary embolism in 10/07. Many of these diagnoses are pathogenetically related to the known Rendu-Osler-Weber disease.

The patient reported that epistaxis first occurred in her adolescence (at the age of 16 years), approximately one to two times per week and self-limiting, but showed a clear progression over the years.

Transfusions were given for the first time at the age of 40 years. At the age of 50 years epistaxis was not any more self-limiting and occurred on average five times a week.

In the last four years she was therefore transfused with more than one hundred erythrocyte concentrates and had partly life-threatening haemoglobin levels. During this period the haemoglobin levels were permanently low with extreme values between 6.3 g/dL and 11.8 g/dL. Although the patient has an arterial hypertension there seemed to be no clear correlation between high blood pressures and severe epistaxis attacks which can be deduced from an excellent documentation by the patients' husband.

The diagnosis Rendu-Osler-Weber disease was identified in the 1980s with a strongly positive family history: her father, two brothers and two of her three children also showed/show symptoms of this illness.

Multiple endoscopic intranasal coagulations were performed using neodymium, CO_2_ lasers, or bipolar coagulation instruments. Failure in the control of the episodes of massive epistaxis led to transarterial embolisation of the maxillary, the facial and the sphenopalatine arteries and attempts of septodermoplasty. Even these extended procedures failed to prevent recurrence.

Two months after extensive neuroradiological embolisation epistaxis was heavier than ever before and the patient became almost entirely dependent on bilateral nasal dressings, so all treatment modalities except radiotherapy were exploited but had no permanent success.

Before we decided to perform an external radiotherapy treatment it was discussed to employ brachytherapy. But as merely contact to the nasal cavity caused heavy bleedings, this modality was not considered as realistic option.

**Figure 1:**
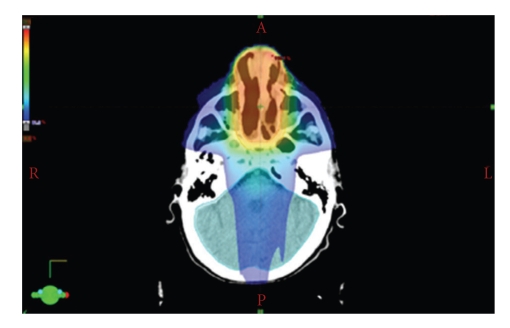
*Radiotherapy planning I*. IMRT plan with the planning target volume (PTV) in one CT cross section, red colours indicate a higher dose; a steep dose gradient can be achieved by using fluence modulation with a multi leaf collimator. Normal tissues are optimally spared.

**Figure 2:**
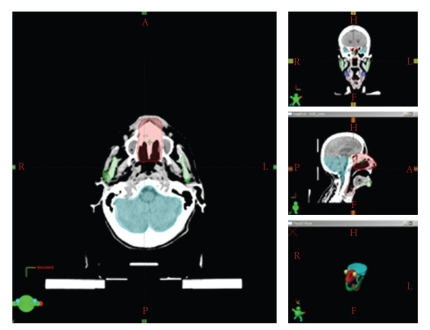
*Radiotherapy planning II*. Another CT section at the lower PTV boundary. Small insets show the complete extension of the PTV.

**Figure 3:**
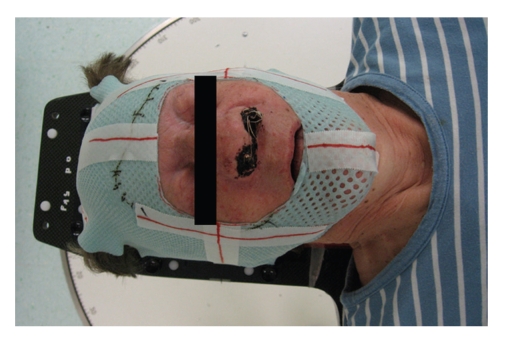
*Radiotherapy positioning.* Positioning of the patient on the couch. The thermoplastic mask system is used to prevent the patient from moving during the treatment. The lines marked on the mask system are used for the room lasers which are used to define the exact positioning.

Radiotherapy planning was performed using the solution commercialized by VARIAN with the Eclipse treatment planning system (TPS) and the Clinac accelerator (Figures 1 and 2). The planning CT was carried out with 3 mm step size, positioning was done with a thermoplastic mask system (Figure 3). Intensity-modulated radiotherapy was performed to optimally spare the surrounding normal tissues. Altogether we administered 50 Gy in 2 Gy single fractions (5 days per week), the therapy lasted five weeks without interruptions. This conventional dosing schedule was chosen to avoid possible late complications concerning the organs at risk, the end dose was specified empirically.

**Figure 4:**
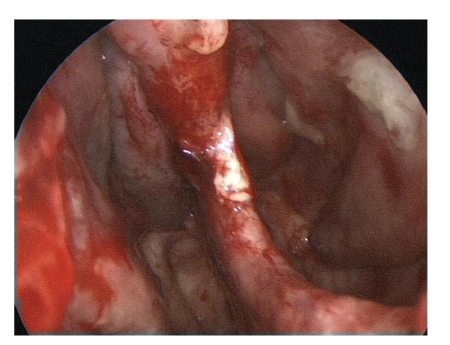
Endoscopy of the right nasal cavity following removal of the nasal dressing 2 weeks after the last radiotherapy showed a vast septal perforation due to multiple endonasal interventions. A 3 mm bluish spot at the dorso apical border of the septal perforation and very fine teleangiectatic vessels were the only possible and now inactive sites of the former extensive teleangiectatic lesions.

**Figure 5:**
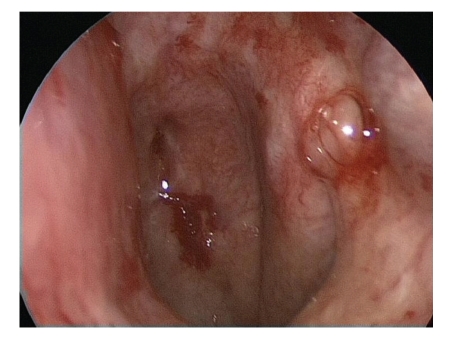
Endoscopic picture of the right nasal cavity with anemic and white nasal mucosa.

The nose was tamponaded before the beginning of the radiotherapy, the tamponade was changed once during the radiotherapy course. To prevent bacterial infections causing sinusitis the patient was daily treated by antibiotics (Aminopenicillin combined with clavulan acid p. o. twice a day). Two weeks after the last radiation fraction the nasal dressing could be removed without problems in the OR. Endoscopical control did reveal an almost avascular white mucosa without any trace of bleeding spots, previously existing hemangiomas and crusts had disappeared. Only on the dorso-apical edge of the vast septal perforation we could detect a 3 mm bluish spot as possible spot for an old hemangioma (Figures 4 and 5).

## 3. Discussion

The rationale to treat HHT-related severe recurrent epistaxis with radiotherapy is to irreversibly destroy fibrodysplastic microvessels. The main mechanism as discussed in the literature might be radiation-induced demise of endothelial cells due to apoptosis. These cells are considered to be very radiosensitive. The exact underlying molecular pathways of endothelium dysfunction are not known so far [7, 8].

A bigger data set was acquired for intranasal brachytherapy for epistaxis in 43 patients with Rendu-Osler disease treated between 1971–1991 at Henri Mondor Hospital. Dose at one application ranged from 15–35 Gy with a median of 30 Gy. The time to recurrence of significant epistaxis ranged from 6–178 months with a median of 24 months. The dose prescribed did not correlate with control rate. The only brachytherapy complication was septal perforation in 4 patients; in one this was a result of repeated nasal coagulation [6]. 

Pizzi et al. performed a similar but smaller study. The dose given to the reference isodose was 30 Gy. Complete remission was seen in 4 patients. In 2 patients the response has lasted 18 and 32 months and 2 others had a shorter follow up. In 5 patients, a good response was obtained (mean: 58 months) [9].

Up to now, only very few trials or case reports are reported on external irradiation [9–11].

Although a bigger data set is missing, external radiotherapy seems to be an effective treatment in this setting, but still has to be used as a second line therapy as other treatment modalities are faster and have potentially less side effects. But as a palliative treatment it may replace other treatment methods, especially if they failed before [9].
